# Autoimmune liver disease-associated serologic profiling in Chinese patients with acute hepatitis E virus infection

**DOI:** 10.1007/s12026-021-09178-4

**Published:** 2021-01-28

**Authors:** Honglian Gui, Weijing Wang, Qing Li, Ziqiang Li, Jie Lu, Qing Xie

**Affiliations:** Department of Infectious Diseases, Ruijin Hospital, Shanghai Jiao Tong University School of Medicine, Shanghai, 200025 China

**Keywords:** Autoimmune hepatitis, Autoantibody, Hepatitis E virus, Liver function, Primary biliary cholangitis

## Abstract

The association between hepatitis E virus (HEV) and autoimmune liver diseases has been well-researched; however, the focus has been on autoimmune hepatitis (AIH) and not primary biliary cholangitis (PBC). Therefore, we aimed to investigate the prevalence and evolution of AIH- and PBC-related autoantibodies in Chinese patients with HEV infection. In this retrospective study, 164 patients with acute HEV were included, specifically those whose liver autoantibody results were available and who had no pre-existing liver disease at the time of HEV diagnosis. Positive liver autoimmune serology was present in 69 (42.1%) patients and 21 (12.8%) had at least two autoantibodies at diagnosis. Greater age and alkaline phosphatase levels were independent risk factors for autoantibody positivity. Follow-up serologic tests, which were available for 27 of the 69 autoantibody-positive patients, showed that although antinuclear antibodies disappeared in 11/20 (55.0%) and antimitochondrial antibodies disappeared in 4/5 (80%) patients, 16 still remained positive for autoantibodies and two of them even developed new PBC-related antibodies, as described below. One patient developed a rim-like ANA pattern, accompanied by an enhancement of anti-gp210 positivity; and the other was diagnosed as PBC, based on chronic elevation of cholestatic enzymes and presentation with de novo AMA-M2, 18 months after HEV clearance. In conclusion, AIH- and PBC-related autoantibodies are frequently present during acute HEV infection, indicating that HEV should be excluded before diagnosing AIH and/or PBC. Importantly, some cases maintained or developed autoantibodies after viral clearance, and one patient subsequently developed PBC, highlighting that these individuals warrant long-term follow-up.

## Introduction

Autoimmune hepatitis (AIH) and primary biliary cholangitis (PBC), two major autoimmune liver diseases, are usually life-threatening when left untreated. Thus, early and reliable diagnoses are paramount for the initiation of appropriate therapy. A detailed characterization of autoantibody specificities and titers is indispensable for the diagnosis of these diseases [1]. In general, the presence of antinuclear antibodies (ANA) and anti-smooth muscle antibodies (SMA) are characteristic of AIH-1 [2], while anti-mitochondrial autoantibodies (AMA) or PBC-specific ANA (mainly including anti-gp210 and anti-sp100) are characteristic of PBC [3].

To date, the etiology of these autoimmune liver diseases has been poorly understood. In addition to genetic predisposition, specific environmental factors have been considered to be triggers for the initiation and/or acceleration of autoimmune processes; these include medications, vaccinations, and infections [4, 5]. Hepatitis viruses that cause direct liver damage have naturally been suspects for the etiology of these diseases. Hepatitis C virus (HCV) has been closely associated with the development of AIH [4, 6, 7].

Hepatitis E, an infectious disease caused by the hepatitis E virus (HEV), has worldwide prevalence [8]. HEV has extensive genetic diversity: HEV genotype (GT)-1 and GT-2 infections are waterborne and causative for epidemics in the tropics, while GT-3 and GT-4 infections are sporadic zoonotic diseases and are mainly transmitted by ingestion of undercooked food [9–11]. The clinical course of these infections differs. GT-1 and GT-2 infections are generally self-limiting and can lead to liver failure with high mortality in pregnant women, while the majority of GT-3 and GT-4 infections have a clinically asymptomatic course and can only cause severe hepatitis and liver failure in the elderly or in patients with underlying liver disease [9]. Immunosuppressed individuals infected with GT-3 or GT-4 may develop chronic hepatitis E, which then leads to rapidly progressing liver disease [12].

With more widespread recognition of HEV, a series of studies has focused on the association between HEV infection and autoimmune liver diseases. A higher HEV seroprevalence has been reported in AIH patients in Germany [13] and in Austria [14], but not in the Netherlands [15], probably due to the fairly high HEV incidence in the latter. Furthermore, some autoantibodies that are typically found in patients with AIH have also been reported in acute hepatitis E patients in Europe, America, and Asia [16–21]. Similarly, the prevalence of anti-HEV antibodies in patients with PBC has also been controversial [22, 23]. Few studies have addressed the presence of PBC-related autoantibodies in patients with acute hepatitis E. One case, positive for both HEV RNA and anti-AMA-M2, was diagnosed as acute HEV infection and confirmed as PBC by liver biopsy [24]. The association between HEV infection and PBC has not been firmly established. Therefore, the aim of the present study was to investigate the prevalence and evolution of AIH- and PBC-related autoantibodies in a well-characterized cohort of Chinese patients with acute HEV infection.

## Patients and methods

The study was conducted in accordance with ethical guidelines of the Declaration of Helsinki 1975 and was approved by the ethics committee of Shanghai Ruijin Hospital (No.2019-251). Written informed consent was exempted on account of retrospective nature of this study.

### Study population

We retrospectively collected information from all consecutive hospitalized patients with acute HEV infection diagnosed at Ruijin Hospital between January 2016 and August 2019. The inclusion criteria were (i) positive HEV RNA in serum and/or serum positive for a combination of anti-HEV IgM and IgG and (ii) liver autoantibody results available at the time of HEV diagnosis. Most patients were diagnosed by HEV serology, since HEV RNA assays had not been used in our hospital until August 2018. No time had elapsed between HEV diagnosis and autoantibody testing, since differential diagnosis was needed immediately for all patients with acute hepatitis of unknown origin. Given that some autoantibodies are not disease-specific and are present in other liver diseases [1], patients with any pre-existing liver disease, including alcoholic/non-alcoholic fatty liver disease (AFLD/NAFLD), chronic hepatitis B and/or hepatitis C, autoimmune liver diseases, and schistosomiasis liver disease, were excluded from the study.

### Study design

All data (demographic, biochemical, and serological features) were extracted from the hospital electronic patient database. Clinical information included age, sex, and history of autoimmune disease, immunosuppression, current malignancies, and pre-existing liver disease. To better represent the severity of disease, the following biochemical data were included: the earliest available alanine aminotransferase (ALT), aspartate aminotransferase (AST), alkaline phosphatase (ALP), and gamma-glutamyl transpeptidase (GGT) levels were recorded, as were the highest available total bilirubin and international normalized ratio (INR) levels, during the whole course of disease. The circulating autoantibodies including AMA, M2, anti-gp210, anti-sp100, ANA, SMA, anti-neutrophil cytoplasmic antibody (ANCA), anti-soluble liver antigen/liver-pancreas antibody (SLA/LP), anti-liver cytosol type 1 antibody (LC-1), and anti-liver-kidney microsomal type 1 antibody (LKM-1) were tested. The follow-up autoantibody and liver function tests, as initiated by the outpatient physicians, were performed using the same methodology as tests at presentation.

### Laboratory assessment

Indirect immunofluorescence assays (IIF) on rat kidney, liver, and stomach tissue (Euroimmun) were performed for detection of liver autoantibodies, and a titer of 1:100 was defined as a positive reaction according to the recommendation of the assay kits. IIF on human epithelial type 2 (HEp2) cells (INOVA) was used for ANA titer determination and pattern definition; its recommended minimum dilution is 1:40 (1:40, 1:80, 1:160, 1:320, etc.). Immunoblotting (with Euroline Liver-Profile 4 IgG from Euroimmun) was performed for SLA/LP, LC-1, LKM-1, anti-sp100, anti-gp210, and anti-M2 antibodies. ANCA was tested by IIF on human granulocytes (Euroimmun), and a titer of 1:10, 1:32, or ≥ 1:100 was defined as a week-positive, positive or strong-positive reaction, respectively; any reactivity was further analyzed using anti-proteinase 3 and anti-myeloperoxidase ELISA kits (Euroimmun). Detection of IgG and IgM HEV-specific antibodies was performed using Wantai HEV ELISA kits. HEV RNA was detected with the Promotor® HEV RNA detection kit (ACON). Serum total immunoglobulin concentrations (including IgG, IgM, IgA, and IgE) were assessed by immunonephelometry. All assays were performed according to the manufacturer’s instructions.

### Statistics

Statistical analysis was performed with IBM SPSS 23 for Windows. Continuous variables were presented as medians (interquartile range, IQR) and compared using Mann-Whitney test. Frequency data were expressed as numbers and percentages and compared using Fisher’s exact test. Variables with a *p* < 0.1 in univariate analysis were further analyzed in multivariate analysis performed by binary logistic regression, with odds ratio (OR) and 95% confidence interval (CI) calculated. *p* values ≤0.05 (two-sided) were considered to be statistically significant.

## Results

### Overview of study population

**Fig. 1:**
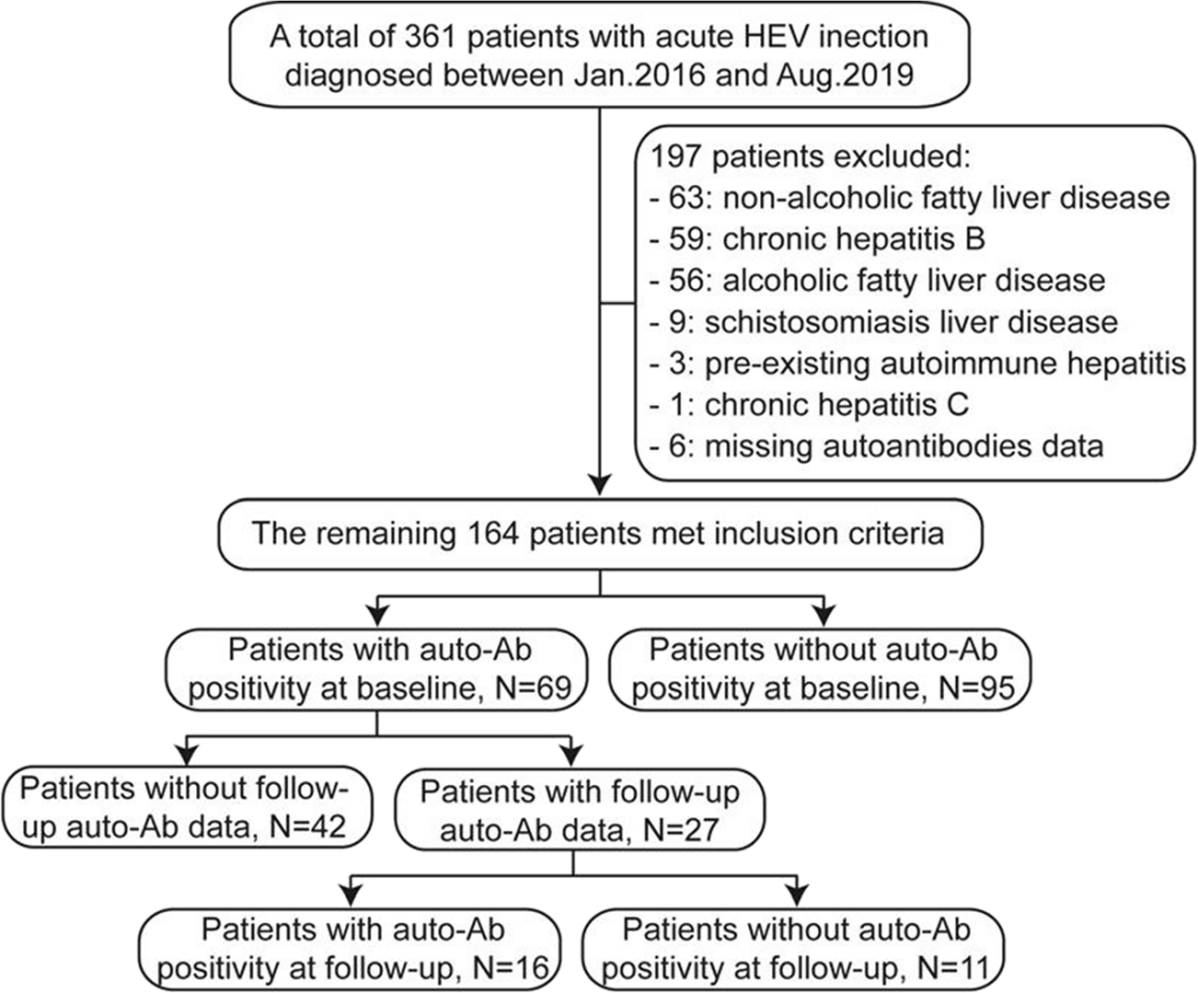
Flow chart illustrating the design

A total of 361 adult patients were diagnosed with acute HEV infection between January 2016 and August 2019, of whom 197 patients were excluded due to the presence of other pre-existing liver diseases or incomplete data on autoantibodies (Fig. 1). The remaining 164 patients met the inclusion criteria, with 93 (56.7%) males and median age at HEV infection of 54 years (range, 25–82). All patients were positive for anti-HEV IgM; HEV RNA was also detected in the serum of 58 patients out of the 62 who were tested. HEV genotyping was successful in 72% of PCR-positive samples (42/58)—29 were classified as 4d, nine were 4b, and one each were 3b, 4a, 4 h, and 4i infections.

Negative HEV RNA results were obtained in all 42 patients who tested positive while in hospital. Notably, eleven patients were taking immunosuppressive drugs at the time of HEV diagnosis, and none of them developed chronic HEV infection: four because of breast cancer (two received epirubicin plus cyclophosphamide; the other two received Herceptin plus docetaxel) and one each because of membranous nephropathy (methylprednisolone plus tacrolimus), nasopharyngeal carcinoma (cisplatin plus 5-fluorouracil, and daily radiation), Behcet’s disease (hydroxychloroquine plus methotrexate), ankylosing spondylitis (recombinant human type II tumor necrosis factor receptor antibody fusion protein for injection 50 mg/month), sicca syndrome (methylprednisolone 2 mg/day), ANCA-associated nephritis (prednisone 30 mg/day), and cutaneous amyloidosis (methylprednisolone 24 mg/day). None of them developed chronic HEV infection and were treated with ribavirin.

Among the 164 patients included in the study, none were treated with ribavirin throughout the course of HEV infection. None underwent a liver biopsy, since the cause of the acute hepatitis was clear, and all patients recovered, except two died during treatment for HEV infection because of complications of severe pulmonary infection and complex abdominal infection, respectively. The other patients had normal liver biochemistry results either when at discharge or at outpatient follow-up within 2 to 4 weeks after discharge (Table 1). Only one patient was re-admitted for persistent liver disease 1.5 years later (described below). No patient was treated with immunosuppressive therapy later since no definitive diagnosis of AIH was made over a short follow-up period in our series.

**Table 1:** Characteristics of patients presenting with or without autoantibodies

	Auto-Abs(+) group at baseline, *n* = 69	Auto-Abs(-) group at baseline, *n* = 95	*p*
Female, *n* (%)	33 (47.8)	38 (40.0)	0.318
Age, y, median (IQR)	61 (50-67)	52 (38-61)	*0.001*
Past/current cancer, *n* (%)	4 (5.8)	7 (7.4)	0.691
Concomitant autoimmune diseases, *n* (%)	11 (15.9)	6 (6.3)	*0.046*
Immunosuppressive treatment, *n* (%)	5 (7.2)	6 (6.3)	0.814
Baseline parameters, median (IQR)			
ALT, ×ULN	31.5 (18.2–43.1)	30.5 (16.2–44.9)	0.794
AST, ×ULN	30.2 (13.8–45.5)	25.8 (10.6–45.8)	0.289
ALP, ×ULN	1.6 (1.2–1.8)	1.3 (1.1–1.7)	*0.009*
GGT, ×ULN	3.1 (2.2–5.0)	2.9 (1.6–4.5)	0.187
Total bilirubin, ×ULN	6.4 (3.5–12.6)	5.0 (1.6–10.3)	0.105
<2 × ULN, *n* (%)	6 (8.7)	25 (26.3)	*0.004*
≥2 × ULN, *n* (%)	63 (91.3)	70 (73.7)	
INR^†^	1.04 (0.96–1.23)	1.03 (0.93–1.17)	0.495
Serum IgG, mg/dL	1475 (1285–1845)	1320 (1190–1550)	*0.006*
Serum IgA, mg/dL	267 (216–351)	241 (196–339)	0.095
Serum IgM, mg/dL	254 (158–375)	195 (137–282)	*0.027*
Serum IgE, IU/mL	82 (30–202)	42 (17–123)	*0.020*
HEV genotype, *n*	19	23	
3b/4a/4b/4d/4 h/4i, *n*	0/1/3/14/1/0	1/0/6/15/0/1	0.543
Follow-up parameters, median (IQR)^**‡**^			
ALT, ×ULN	0.3 (0.2–0.4)	0.3 (0.3–0.5)	0.078
AST, ×ULN	0.6 (0.5–0.7)	0.6 (0.5–0.7)	0.109
ALP, ×ULN	0.7 (0.5–0.8)	0.6 (0.5–0.7)	0.748
GGT, ×ULN	0.5 (0.3–0.7)	0.5 (0.3–0.8)	0.748
Total bilirubin, ×ULN	0.8 (0.6–0.9)	0.8 (0.7–1.0)	0.142

^†^Two patients were excluded because of taking anticoagulant warfarin

^**‡**^Two patients were excluded due to death

*ALT* alanine aminotransferase, *ALP* alkaline phosphatase, *AST* aspartate aminotransferase, *HEV* hepatitis E virus, *Ig* immunoglobulin, *INR* International Normalized Ratio, *IQR* interquartile range, *GGT* gamma-glutamyl transpeptidase, *ULN* upper limit of normal

### Liver autoimmune serology at acute HEV infection

Positive liver autoimmune serology was present in 69 (42.1%) of the patients at the time of acute HEV infection, and 21 (12.8%) had two or more autoantibodies detected.

**Fig. 2:**
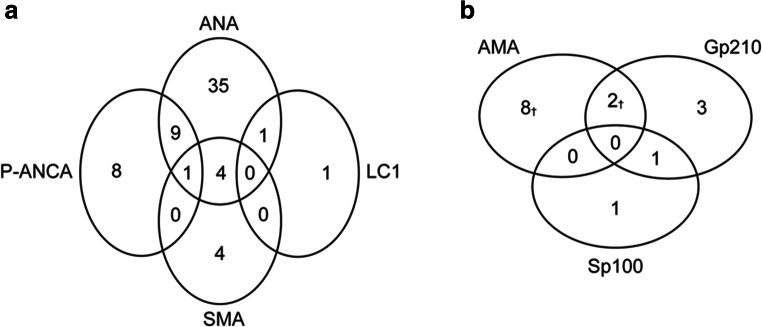
Numbers of acute hepatitis E patients with positive AIH- or PBC-associated autoantibodies. Patients were positive for one or more of the following antibodies: **a** AIH-associated autoantibodies; **b** PBC-associated autoantibodies, detected at the time of acute HEV infection. ^**†**^One patient each presented with M2-subtype positivity; ^**‡**^nine patients had antibodies for both AIH and PBC

AIH-associated autoantibodies were detected in 63 patients (Fig. 2a). ANA tests were positive in 50 patients (30.5%), with titers ranging from 1:80 to 1:320 (22 multiple nuclear dots (MND), 11 speckled, eight homogeneous, four nucleolar, and five mixed patterns). SMA was positive in nine patients (5.5%). Two patients (1.2%) had positive immunoblotting for anti-LC1: one case was a 36-year-old male with anti-LC1 as the only serological marker; the other was a 56-year-old female, accompanied by mixed IIF pattern for ANA and positive immunoblotting for anti-gp210 and anti-sp100, with rapid normalization of the transaminase levels. Anti-SLA and LKM1 were negative in all patients. P-ANCA was detected in 18 patients (13 weakly positive, four positive, one strongly positive) and appeared alone in eight patients, alongside ANA in nine, and together with both ANA and SMA in one. Sera positive for ANCA at IIF on human granulocytes did not react with any of the main molecular ANCA targets as assessed by ELISAs.

PBC-associated autoantibodies were detected in 15 patients (Fig. 2b). IIF-AMA positivity on triple rodent tissue was detected in 10 patients (6.1%); of whom 3 were M2 subtype positive when tested by immunoblotting, while the 7 remaining were negative when tested by immunoblotting, showing an atypical AMA-positive immunofluorescence, but negative immunoblot. Anti-gp210 was detected in 6 patients (two positive, four weakly positive); 5 of them were also positive for ANA (1 homogeneous, 2 speckled, and 2 mixed patterns (rim-like accompanied by MND and homogeneous pattern, respectively)). Anti-sp100, another PBC-specific ANA, was present in two patients; both were also positive for ANA (1 MND and 1 mixed patterns). There were nine patients positive for both AIH- and PBC-related antibodies.

As shown in Table 1, the patients with autoantibody positivity were of a significantly greater age, had more concomitant autoimmune diseases and higher incidence of clinical jaundice, and elevated levels of serum alkaline phosphatase (ALP), IgG, IgM, and IgE than patients with no autoantibody detectable at the time of acute HEV infection. In a further multivariate analysis, greater age (OR 0.968, 95% CI: 0.941–0.997, *p* = 0.030) and ALP levels (OR 0.995, 95%CI: 0.989–1.000, *p* = 0.043) were independent risk factors for positivity for autoantibodies associated with AIH and/or PBC in patients with acute HEV infection.

### Prevalence of autoantibodies at follow-up

Twenty-seven of the 69 autoantibody-positive patients at the time of acute HEV infection had follow-up data available for autoantibodies. Autoantibodies disappeared in 11 patients at a follow-up of 2 to 6 months, while autoantibodies were still present in the other 16 patients at a median (IQR) 14 (8–18) months of follow-up. As shown in Table 2, the patients whose autoantibodies became negative had higher serum IgE levels at baseline when compared with the patients who had remained positive for autoantibodies at follow-up.

**Table 2:** Clinical features between two subgroups with different evolution of autoantibodies

	Auto-Ab(+) subgroup at follow-up, *n* = 16	Auto-Ab(-) subgroup at follow-up, *n* = 11	*p*
Female, *n* (%)	9 (56.3)	5 (45.5)	0.704
Age, y, median (IQR)	61 (58–68)	62 (41–66)	0.521
Concomitant autoimmune diseases, *n* (%)	5 (31.3)	3 (27.3)	0.100
Baseline parameters, median (IQR)			
ALT, ×ULN	32.6 (23.1–42.7)	30.9 (19.6–48.8)	0.961
AST, ×ULN	33.0 (18.6–43.5)	43.0 (16.8–52.2)	0.730
ALP, ×ULN	1.6 (1.4–1.7)	1.5 (1.4–2.1)	0.882
GGT, ×ULN	3.1 (1.9–4.4)	3.1 (2.2–4.2)	0.554
Total bilirubin, ×ULN	6.5 (5.4–9.2)	10.0 (3.7–12.7)	0.554
INR	1.02 (0.95–1.16)	1.03 (0.95–1.14)	0.586
Serum IgG, mg/dL	1550 (1255–1645)	1440 (1290–1575)	0.748
Serum IgA, mg/dL	304 (267–346)	302 (211–355)	0.537
Serum IgM, mg/dL	239 (175–269)	325 (158–356)	0.622
Serum IgE, IU/mL	39 (17–103)	127 (66–225)	*0.046*
Follow-up parameters, median (IQR)			
ALT, ×ULN	0.3 (0.2–0.4)	0.3 (0.2–0.3)	0.334
AST, ×ULN	0.6 (0.5–0.6)	0.5 (0.5–0.6)	0.308
ALP, ×ULN	0.6 (0.5–0.8)	0.6 (0.5–0.9)	0.980
GGT, ×ULN	0.3 (0.2–0.4)	0.6 (0.3–0.7)	0.108
Total bilirubin, ×ULN	0.7 (0.6–0.8)	0.8 (0.7–0.9)	0.570

*HEV* hepatitis E virus, *IQR* interquartile range, *ALT* alanine aminotransferase, *ULN* upper limit of normal, *AST* aspartate aminotransferase, *ALP* alkaline phosphatase, *GGT* gamma-glutamyl transpeptidase, *INR* International Normalized Ratio, *Ig* immunoglobulin

**Fig. 3:**
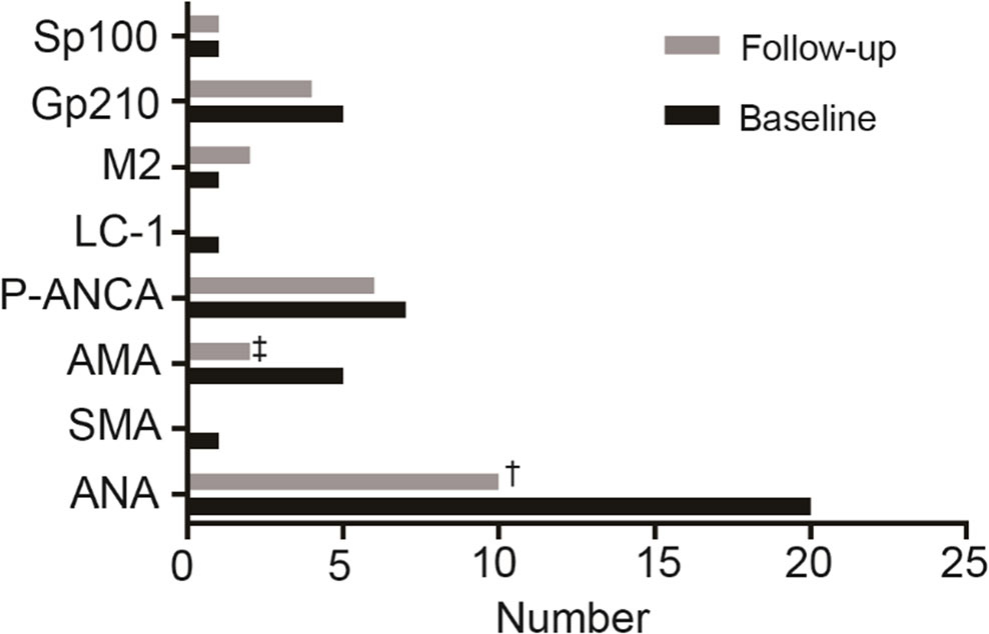
Alteration of autoantibody profiles from baseline to follow-up. ^**†**^One patient developed a rim-like ANA pattern, so ANA actually disappeared in 11 of 20 (55.0%); ^**‡**^one patient developed de novo AMA, so AMA actually disappeared in 4 of 5 (80.0%)

Alterations in autoantibody profiles from baseline to follow-up are shown in Fig. 3. ANA disappeared in 11 of 20 (55.0%) donors (6 out of 9 MND, 3 out of 5 speckled, and 2 out of 2 nucleolar); and a rim-like ANA pattern with a titer of 1:80 developed two months after HEV clearance (see case A). In the older female patient, anti-LC1 disappeared 3 months after HEV clearance, but positivity for PBC-specific ANA anti-sp100 and anti-gp210 remained 1 year after discharge. Among five patients with AMA IIF positivity, four did not show M2 subtype positive when tested by immunoblotting and became negative after HEV clearance; the other patient with AMA-M2 subtype was still positive 18 months later, but was not associated with biochemical cholestasis. However, one developed de novo AMA-M2 18 months after HEV clearance and was diagnosed with PBC (see case B).

### Case presentations

Case A was a 61-year-old female. She presented weakly positive for anti-gp210 by immunoblotting and negative for ANA at diagnosis with acute hepatitis E. She developed a new antibody, showing a rim-like ANA pattern with a titer of 1:80, accompanied by positive immunoblotting for anti-gp210, 2 months after discharge. She maintained positivity for rim-like ANA and anti-gp210 1 year after discharge, but with no evidence of PBC (lack of symptoms, normal liver enzymes, normal abdomen ultrasound, and liver stiffness).

Case B was a 67-year-old male. He presented with a speckled-like ANA with a titer of 1:80 and negative AMA at diagnosis with acute hepatitis E. After 2 weeks of treatment, he was discharged with normalization of all liver function assays. Liver biochemical tests were normal 6 months after discharge. Liver biochemical tests were abnormal 1 year after discharge (ALT 58 IU/L, AST 59 IU/L, ALP 247 IU/L, GGT 671 IU/L), and a computed tomography scan of the abdomen showed no biliary obstruction. A further 6 months later, he had raised cholestatic enzymes (ALP 358 IU/L, GGT 961 IU/L), accompanied by the presence of speckled-like ANA with a titer of 1:160, de novo AMA by IIF and M2 by immunoblotting. Finally, he was diagnosed with PBC 1.5 years after HEV clearance and commenced ursodeoxycholic acid (UDCA, 13-15 mg/kg/d) treatment. However, he had abnormal liver biochemical tests (ALT 18 IU/L, AST 37 IU/L, ALP 235 IU/L, GGT 354 IU/L, and total bilirubin 10.3 μmol/L) and an inadequate biochemical response to UDCA, according the Paris 2 criteria [25], 12 months after treatment.

## Discussion

By analyzing liver autoantibodies in a cohort of adult Chinese patients with acute HEV infection, our results indicated that a high proportion of hepatitis E patients were positive for AIH- and PBC-related autoantibodies at the time of HEV infection. Viral infection may trigger autoimmunity in susceptible individuals. Cross-reactivity between immunogenic viral epitopes and auto-antigens has been characterized for many pathogens [4, 16]. Clearance of viremia would be expected to be accompanied by disappearance of these cross-reactive autoantibodies, as indeed was seen in a considerable proportion of patients in this study. However, 45% of ANA-positive and 20% of AMA-positive patients still remained positive at extended time-points; two patients (case A and B) even developed de novo PBC-related autoantibodies. Early encounter with HCV has been shown to trigger late autoimmune mechanisms [4, 6], which might also be the case for HEV.

Notably, 6.1% of our patients were positive for AMA, which was higher than reported in other studies [19–21]. Admittedly, it is difficult to ascertain whether AMA positivity was secondary to previously undiagnosed PBC or HEV-mediated seroconversion. In our study, 7 out of 10 AMA-positive patients were not confirmed by molecular testing at presentation; and AMA positivity disappeared in all four cases who had follow-up antibodies after HEV clearance. The atypical AMA had a positive immunofluorescence pattern, and staining revealed that they were present in all renal tubules, gastric parietal cells, and hepatocyte cytoplasm and had a negative immunoblot which is a specific characteristic of AMA in PBC, as was described previously [19]. Atypical AMAs were also not associated with biochemical cholestasis. Transient positivity for atypical AMA might be due to acute liver injury, which usually disappears with the resolution of acute disease [20, 26].

ANA is not specific for AIH or PBC; ANA positivity is still considered as a classification criterion for the diagnosis of autoimmune diseases, used to classify patients with AIH-1. The majority of AIH-1 patients show a homogeneous pattern, and speckled or nucleolar patterns are also encountered, while ANA with either MND or rim-like membranous pattern are highly specific for PBC [2]. In our study, homogeneous pattern, which only accounted for a small part of ANA positivity, persisted. Surprisingly, MND was the most frequent ANA pattern, with only two confirmed by anti-sp100 immunoblotting; 2/3 of them then disappeared at follow-up. In fact, MND, differentiated by anti-sp100, can also be found in other diseases, e.g., rheumatological and other connective tissue disorders, while anti-sp100 is much more specific for PBC [27, 28]. The exact reason for the high number of patients with ANA MND pattern is unclear and worthy of further investigations.

Of note, a high proportion of AMA and/or PBC-specific ANA-positive patients with normal liver enzymes have been shown to have liver pathology consistent with PBC and to ultimately progress over time to develop overt clinical and biochemical features of PBC [3, 29, 30]. As mentioned, the diagnosis of PBC requires at least two out of the three criteria—specific autoantibody, elevated cholestatic enzymes, and histological findings [3]. Because histological diagnosis was missing for some patients with AMA and/or PBC-specific ANA seropositivity, the possibility exists that the patient who ultimately developed PBC was already progressing towards PBC before or at the time of HEV infection. We were unable to establish an association of PBC with HEV infection, as only one or two patients developed de novo autoantibodies after HEV resolution. However, the presence of HEV that has been reported in hepatocytes and bile duct epithelia of chronic HEV patients seems to indicate that HEV causes bile duct injury [31].

Importantly, this study represents an attempt to identify risk factors for autoantibody positivity in patients with acute HEV infection. Our results showed that the prevalence of autoantibodies increases with age, which may reflect cumulative exposure to environmental factors, as in the case of ANA [32, 33]. Notably, high levels of ALP were observed in acute HEV patients with autoantibodies. One possible explanation is that liver disease caused by HEV infection may be partly due to bile duct-related immune responses, including autoimmunity [34]. Increased levels of serum IgG and IgM have been shown to be distinctive biochemical features of AIH and PBC, respectively [35, 36]. Higher serum IgE levels were observed to more likely occur in patients with positive autoantibodies at the time of acute HEV infection, and autoantibodies disappeared after HEV clearance. These results suggest that the hypersensitivity reaction initiated by antiviral immunity could be the major cause of the pathologic manifestations of viral disease. As only a few of the total serum Ig levels were determined at follow up, it was difficult to follow the evolution of Ig titers. As 67% of the study population was infected with subtype 4d, no significant association between HEV genotype and evolution of autoantibodies was observed. Our study and that of Wu et al. [21] identified completely different risk factors for autoantibody positivity. This might have been caused by different clinical phenotypes of acute HEV infection in the two cohorts. For example, there were more aminotransferases and less bilirubin in the present cohort compared to that of Wu et al.

This study had several limitations. Firstly, the current study, as for Terziroli et al. [19] and Wu et al. [21], did not incorporate a control group to compare background levels of autoantibodies, e.g., age- and sex-matched healthy controls. Secondly, the autoantibody-negative patients at presentation were not followed up, which introduces potential bias when examining longitudinal changes in autoantibodies, since it remains unclear if those patients developed autoantibodies after clearing HEV. Recently, one study of HCV reported that 27% of those who were negative pre-treatment developed de novo autoantibodies after cure of HCV by direct-acting antiviral agents [6]. Thirdly, the cut-off for IIF on triple rodent tissue used here was 1:100, higher than the one recommended, and the IIF pattern of SMA was unspecified on the records. Therefore, a prospective study with more patients with HEV infection is required to validate longitudinal changes in autoantibodies, especially for those autoantibody-negative patients, using the latest testing standard [2].

In conclusion, our findings indicate that autoantibodies associated with AIH or PBC are frequently present during acute HEV infection, indicating that HEV infection should be excluded before diagnosing AIH and/or PBC. Importantly, in some cases, these autoantibodies persist after viral clearance. Further, autoimmune liver disease may be more prominent during extended time periods, since one of our cases developed de novo autoantibodies and progressed to PBC in the medium-term. Our research suggests that the presence of autoantibodies may be considered as an early warning of preclinical autoimmunity and may identify the need for long-term follow-up.

## Data Availability

The datasets generated and analyzed during the present study are available from the corresponding author on reasonable request.
